# The technology of artificial pneumoperitoneum CT and its application in diagnosis of abdominal adhesion

**DOI:** 10.1038/s41598-021-00408-1

**Published:** 2021-10-21

**Authors:** Gui-sheng Wang, Zhi-yi Zhang, Xue-ting Qi, Jin Liu, Ting Liu, Jing-wei Zhao, Xiao-xia Chen, Yi Chen

**Affiliations:** 1 Department of CT, The Third Medical Center of Chinese PLA General Hospital, Beijing, 100089 China; 2 Center of PET-CT, 920th Hospital of Joint Logistics Support Force, Kunming, 650032 China

**Keywords:** Intestinal diseases, Gastrointestinal diseases

## Abstract

To retrospectively analyze the use of artificial pneumoperitoneum in CT scans, to explore its operation methods and technical points, and to lay the foundation for the widespread application of artificial pneumoperitoneum in CT. A total of 331 patients who underwent artificial pneumoperitoneum with CT angiography from January 1, 2013, to November 1, 2019, were recruited. All patients underwent standardized artificial pneumoperitoneum in the horizontal, left and right lateral, and prone positions during CT thin-layer scans of the abdomen and 3D reconstruction. Taking the surgical results as the gold standard, and using kappa test to verify the consistency of surgical results and imaging results. In all 331 patients, 43 patients had a normal peritoneal space, and 288 patients had an abnormal peritoneal space. And only 22 patients developed complications of subcutaneous emphysema, accounting for 6.6% of all 331 patients. In terms of the postoperative results, 28 were normal, and 303 were abnormal. The sensitivity, specificity and accuracy of CT diagnosis of abdominal adhesions using artificial pneumoperitoneum were 100%, 95.04%, and 95.46%, respectively. According to the Kappa consistency test, the imaging diagnosis from the CT scan with artificial pneumoperitoneum had a high consistency with the surgical results (kappa = 0.796, *P* < 0.05). The technique of artificial pneumoperitoneum CT is safe, reliable, highly practical, and proficient for obtaining good imaging results. It provides a good imaging basis for the diagnosis of intra-abdominal diseases, especially intra-abdominal adhesions.

## Introduction

Intra-abdominal adhesion is the abnormal adhesion of abdominal organs to the abdominal wall or the peritoneum. It does not involve changes in organs. It is a common result of intra-abdominal inflammatory reactions, injuries, infections and ischaemia. Since adhesion pathology does not involve volume changes and the physical and chemical properties of the surrounding tissues are the same, the imaging examination of intra-abdominal adhesions is very difficult, and it can only rely on indirect imaging signs such as local intestinal wall thickening, a relatively fixed intestinal position, and panniculitis^1,2^; on the other hand, exploratory laparotomy and laparoscopy can be used to confirm the diagnosis.

Artificial pneumoperitoneum was originally used in laparoscopic surgery to separate the abdominal wall from the abdominal organs with the help of gas pressure, providing surgeons with a wide field of vision and the space to easily perform operations. With the development of CT (Computed tomography), artificial pneumoperitoneum has gradually been used as an imaging method during CT scanning. The use of CT scans with artificial pneumoperitoneum can obtain the tomographic anatomy of abdominal internal organs and the peritoneal space with imaging, providing a more intuitive basis for the diagnosis and treatment of intra-abdominal diseases. At present, the diagnosis of intra-abdominal diseases, especially intestinal adhesions, is mainly based on clinical symptoms, and the diagnosis is confirmed through surgery or laparoscopy. Conventional abdominal CT scans have a poor ability to display information pertaining to intra-abdominal adhesions. The technology of artificial pneumoperitoneum in contrast CT provides a new avenue for the diagnosis and treatment of abdominal adhesions. Providing imaging data for intra-abdominal adhesions before surgery and clarifying the location and severity of adhesions may help reduce intraoperative risks. Based on our hospital’s characteristic diagnosis and treatment technology, in this study, artificial pneumoperitoneum CT examinations were performed in patients with high clinical suspicion of intra-abdominal adhesions. We have accumulated rich experience in the technology of artificial pneumoperitoneum CT. Herein, we will summarize this method of artificial pneumoperitoneum CT and related technical points.

## Methods

### Ethical approval

This study was approved by the Ethics Committee of the Third Medical Center of Chinese PLA General Hospital. The patient’s informed consent was obtained.

### Clinical characteristics

*Inclusion criteria* (1) Patients with high suspicion of abdominal adhesions in clinical diagnosis, such as small bowel obstruction and chronic abdominal complaints; (2) Complete CT scan of pneumoperitoneum; (3) Complete surgical records;

*Exclusion criteria* (1) patients who cannot tolerate and have not undergone complete pneumoperitoneum; (2) patients who have not undergone surgery; (3) the image quality is poor and the diagnosis cannot be completed;

We collected a total of 416 patients from December 2013 to June 2019, finally, 331 patients were included in the study, the information of patients are shown in Table 1.

**Table 1:** The general information of 331 patients.

Characteristic	Patients
Number of patients	331
Age, years (range)	43 (12–76)
Sex	Male 150 (45.31%)
	Female 181 (54.68%)
CTDI vol (mGy)	6.36 ± 2.38
Image results	Normal 43
	Abnormal 288
Operation results	Normal 28
	Abnormal 303

### Examination

#### Artificial pneumoperitoneum procedure

The patient’s legs were flexed to relieve tension on the abdominal wall. A 14 G trocar (B. Braun Introcan Safety IV Catheter) was used to puncture and enter the abdominal cavity in Mc Burney and anti-Mc Burney. The injection of a small amount of air without resistance and without a patient pain response (if a patient felt pain with the injection of a small amount of air, the subcutaneous tissue space may have been punctured, causing subcutaneous emphysema) was considered preliminary confirmation of entering the abdominal cavity. The metal needle core was removed while maintaining the plastic outer tube, a gas injection catheter was fixed to a large-volume syringe or gas injection balloon, and the injection of sterile CO_2_ was initiated into the abdominal cavity. At the same time, the pressure in the abdominal cavity is measured by connecting the micro pressure gauge through the three-way stopcock. The initial insufflation into the abdominal cavity was performed slowly, and the inflation rate could be increased after successful pneumoperitoneum was confirmed. If successful pneumoperitoneum was unclear, success could be determined with X-ray fluoroscopy in the head-high and half-slope positions. Generally, the gas injection process took approximately several minutes, the gas injection volume was usually 2000–3000 ml, and the intra-abdominal pressure measured by the micromanometer was usually 7–8 mmHg.

#### The methods of CT and reconstruction

After the injection of gas was completed, a CT scan was performed quickly, and the abdomen was scanned with a Siemens dual-source Flash CT machine. The layer thickness was 1.25 mm, the pitch was 1.375, the voltage was 120 kV, the current was 35 mA, and the speed was 0.8 s/r. The patient was placed in the supine position, the left and right lateral positions and the prone position for scanning, and the scanning range was from the top of the diaphragm to the pelvic floor. The appropriate window width and window level were selected according to different observation purposes, and for image post-processing, the volume rendering (VR) method was adopted. After the scan was complete, the gas in the abdomen could be drained. The abdominal wall collapsed and recovered within a few seconds if gas exhaust was smooth. If there was additional residual gas, the patient position was adjusted and the abdominal wall was compressed to help remove the residual gas in the abdomen. A small amount of gas was absorbed on its own within 1–2 days.

### Image data and clinical data processing

The imaging data were all jointly read by three doctors with the title of attending physician or above who were engaged in the abdominal imaging diagnosis, and they reached a consensus through consultation. The surgical result was obtained from patient medical records and surgical videos. And the surgical result was taken as the gold standard and compared with the diagnostic result of artificial pneumoperitoneum CT.

Taking the surgical results as the gold standard, and through retrospective analysis of the imaging data of previous cases, we will classify the severity of the patient’s abdominal cavity after CT examination of pneumoperitoneum: level 0: no obvious abnormalities; level 1: bowel or Omentum structure and abdominal wall showed single-point adhesion, or simple intestinal adhesion; level 2: Intestinal tube and (or) omentum structure and abdominal wall showed multi-point adhesion; level 3: Intestine and (or) omentum structure and abdominal wall were diffuse Adhesion.

### Statistical analysis

SPSS 23.0 statistical software was used for data analysis, and the consistency of the image diagnostic results and surgical results after pneumoperitoneum was evaluated with a Kappa test. Kappa > 0.75 indicated high consistency, < 0.40 indicated poor consistency, and 0.40–0.75 indicated fair consistency.

### Statement

All methods were performed in accordance with the relevant guidelines and regulations.

## Results

### Normal pneumoperitoneal space

**Figure 1:**
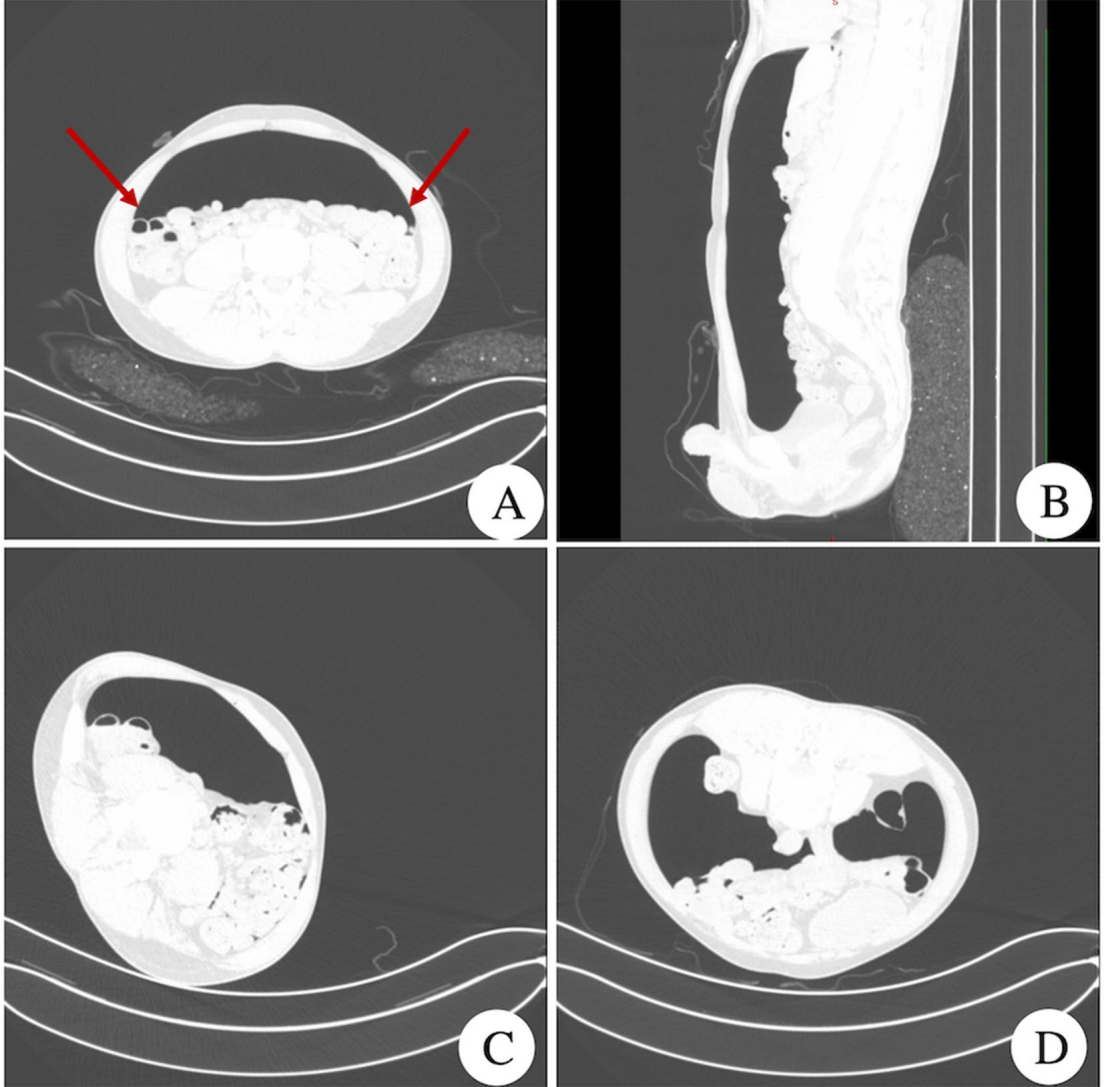
Normal pneumoperitoneal space. (**A**) Shows a normal horizontal axial artificial pneumoperitoneal image (lung window). The peritoneum of the visceral wall is separated, and there is a half-moon-shaped pneumoperitoneal space. The arrows show that the paracolic sulci on both sides are clear and sharp. (**B**) Shows a normal sagittal image of artificial pneumoperitoneum. The pneumoperitoneal space has the shape of a long knife, and the peritoneum of the visceral wall is clearly separated. (**C**) Shows the image of a normal oblique artificial pneumoperitoneum, the abdominal organs are tilted to one side, the contralateral pneumoperitoneum is clear, and the abdominal organs and lateral abdominal wall are clearly displayed. (**D**) Shows the image of the artificial pneumoperitoneum in the normal prone position. Gas fills the space between the posterior abdominal wall and the visceral peritoneum. The relationship between the abdominal organs and the posterior abdominal wall is clearly shown.

A total of 43 patients showed a normal pneumoperitoneal space, of whom 15 patients were confirmed by surgery to have abdominal adhesions. The axial view of the normal artificial pneumoperitoneal image showed a bulging abdomen; the visceral peritoneum was separated from the peritoneum of the abdominal wall, the peritoneal cavity was full of gas, the pneumoperitoneal space was clearly displayed, the abdominal wall was thin and uniform, and the inner surface was smooth. The internal organs of the abdominal cavity were spread out evenly. There was a broad semi-moon-shaped pneumoperitoneal space between the abdominal internal organs and the anterior abdominal wall. The intestinal tube was not expanded, and the wall was thin and evenly circular. The sagittally reconstructed image showed that the pneumoperitoneum had the shape of a long knife. Oblique images of the normal artificial pneumoperitoneum showed that the abdominal bowel tended towards one side under the action of gravity. The space between the bowel and the oblique side of the abdominal wall was increased due to the filling of gas. At this time, the relationship between the bowel and the lateral abdominal wall was clearly displayed. In the prone position, the abdominal internal organs were in close contact with the anterior abdominal wall under the action of gravity, and air filled the space between the posterior abdominal wall and the visceral peritoneum. The abdominal internal organs were separated from the posterior peritoneum. At this time, the relationship between the abdominal internal organs and the posterior abdominal wall was clearly displayed (Fig. 1).

### Abnormal pneumoperitoneal space

**Figure 2:**
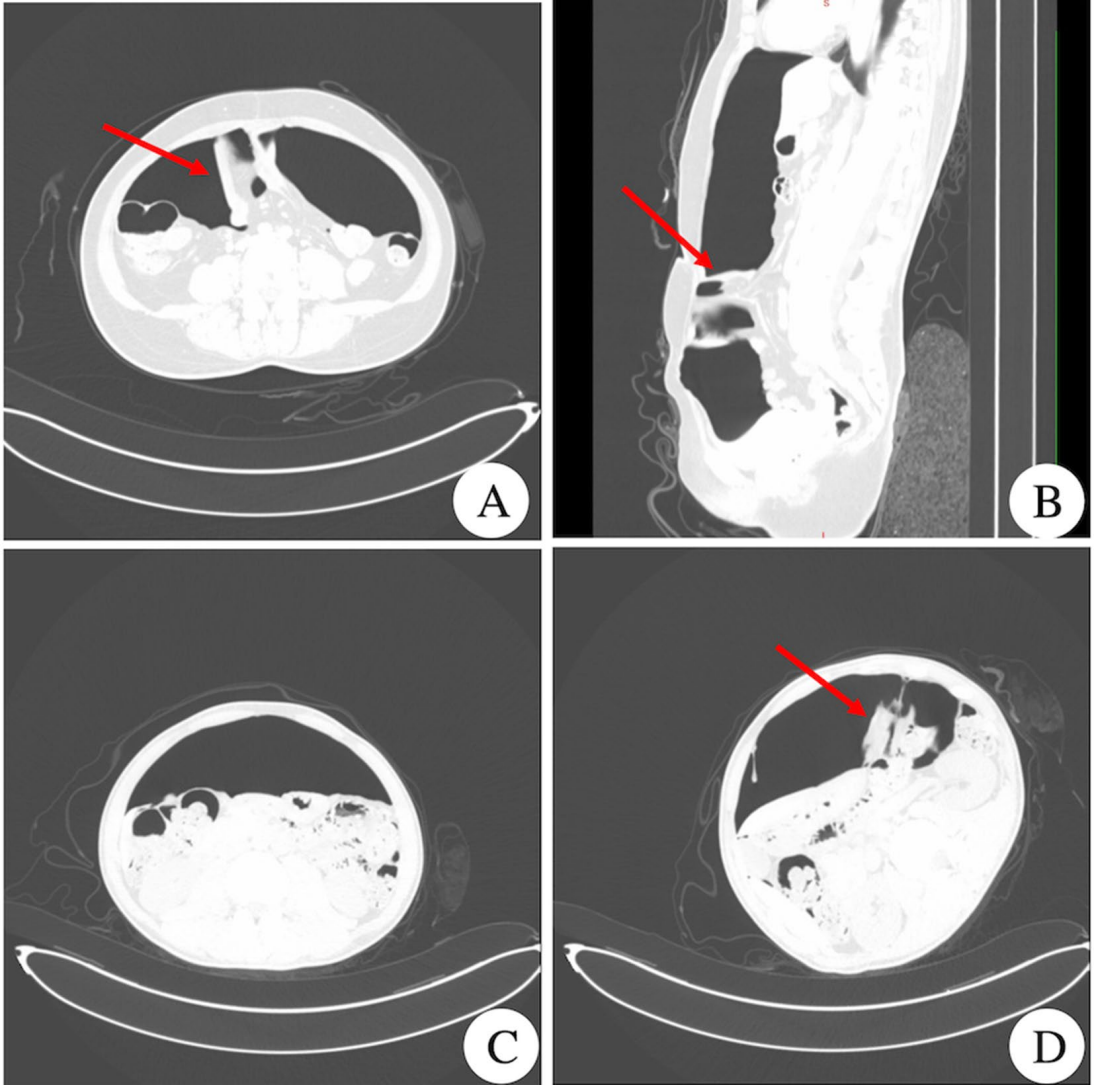
Abnormal pneumoperitoneal space, (**A**) and (**B**) are axial and sagittal pneumoperitoneal images from the same patient. The arrow in the figure shows the small intestine and mesenteric structure that spans the pneumoperitoneal space and connects to the abdominal wall and the visceral plane; (**C**) shows the appearance of a normal pneumoperitoneal space; (**D**) is an image at the same level from the same patient as in (**C**). As shown by the arrow, adhesions of the abdominal organs and left abdominal wall can be seen.

A total of 288 patients showed an abnormal pneumoperitoneal space, which was confirmed by surgery to have adhesions in the abdominal cavity. The abnormal pneumoperitoneal space was characterized by structural adhesions that spanned the pneumoperitoneal space and connected to the abdominal wall and the visceral plane. The adhesions could be divided into regional limitation adhesions and adhesions according to their distribution and extent. Direct abdominal adhesions could be single or multiple loops of bowel, omentum or other organs, such as the liver or uterus, and could be re-adhered to other bowel belt cords in these tissue formations, and a narrow width was formed in the conical body. Sagittal reconstruction was particularly suitable for displaying the continuous hierarchical structure of wall-like adhesions (Fig. 2).

### Image reconstruction

**Figure 3:**
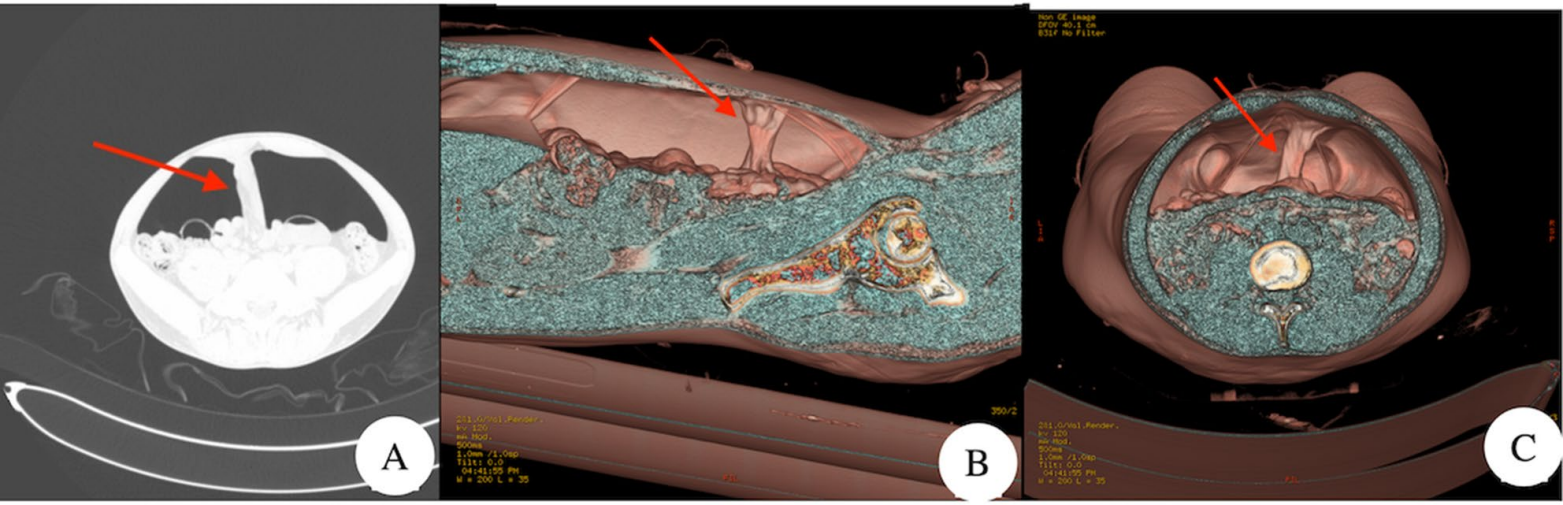
Image reconstruction, (**B**) and (**C**) are reconstructed on the basis of (**A**), the arrow shows the adhesions of intestine and abdominal wall.

By using the VR method to reconstruct the image, the two-dimensional image can be converted into a three-dimensional image. For the surgeon, ordinary two-dimensional images may not be familiar. Through image reconstruction, the surgeon can also understand the abdominal cavity more intuitively. It is very helpful for planning the surgical path before surgery (Fig. 3).

### Comparison of imaging diagnostic results and postoperative results

**Figure 4:**
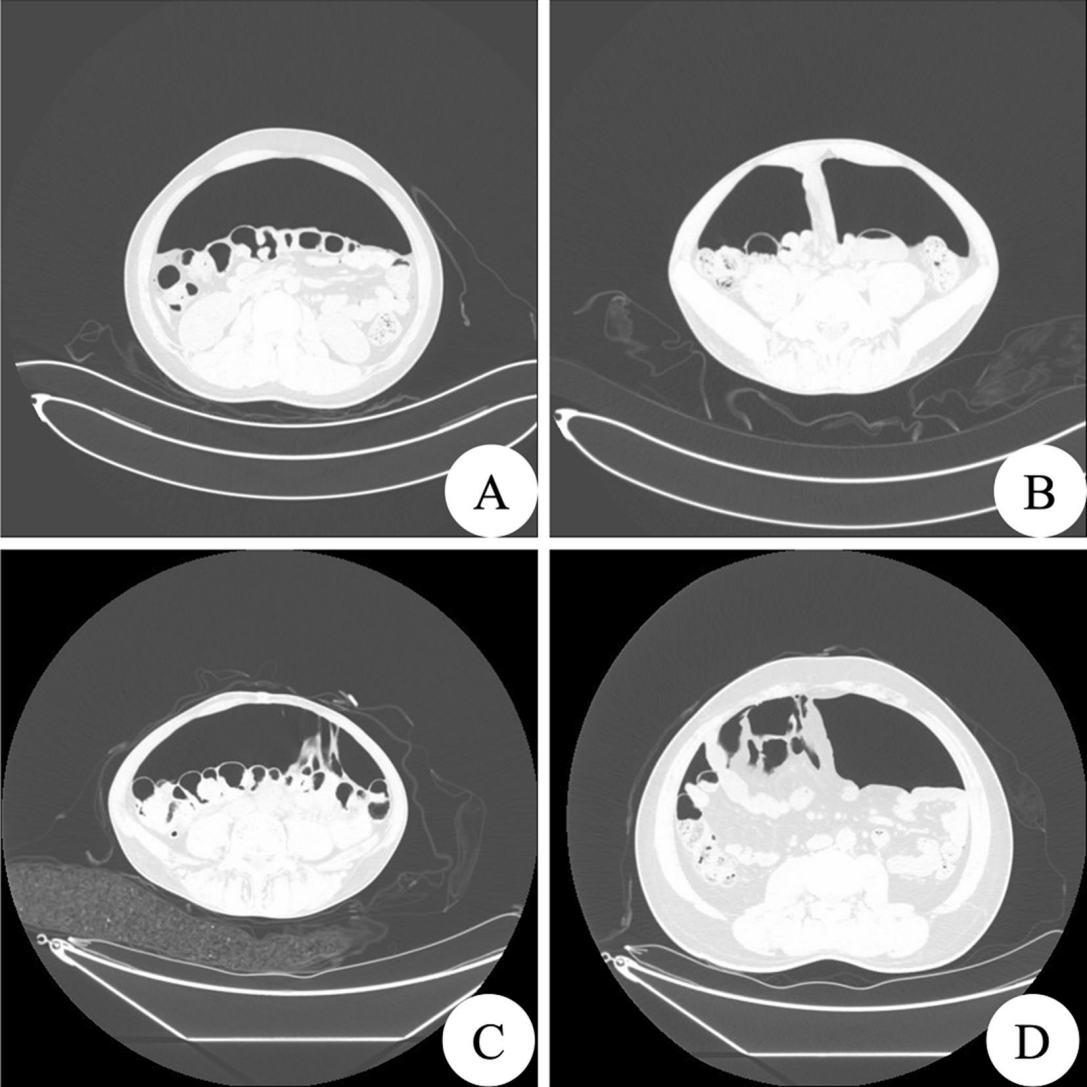
Image of the graded diagnosis of intestinal adhesions on CT with pneumoperitoneum. (**A**) Shows the CT image of the abdomen after pneumoperitoneum as level 0. (**B**) Shows the CT grade diagnosis of pneumoperitoneum as level 1. There is a single point adhesion between the local small bowel tube and the anterior abdominal wall. (**C**) Shows that the CT classification of pneumoperitoneum is level 2, showing that part of the intestinal tube and omentum tissue has multiple adhesions to the anterior abdominal wall. (**D**) Shows that the CT classification of pneumoperitoneum is level 3, and some diffuse adhesions of the anterior abdominal wall of the intestine and omentum tissue can be seen.

The CT imaging diagnostic results of artificial pneumoperitoneum were compared with the surgical results (Table 2). The image standard for graded diagnosis is shown in Fig. 4. The sensitivity, specificity and accuracy of each level of diagnosis are shown in Table 3. The sensitivity, specificity and accuracy of CT diagnosis of abdominal adhesions using artificial pneumoperitoneum were 100%, 95.04%, and 95.46%, respectively. After the kappa test, the CT imaging diagnostic results of pneumoperitoneum were highly consistent with the surgical results (*P* < 0.05, kappa = 0.796).

**Table 2:** Comparison of CT imaging diagnostic results of pneumoperitoneum and operation results.

CT imaging diagnostic results of pneumoperitoneum	Operation results				Total
	0	1	2	3	
0	28	15	0	0	43
1	0	44	13	0	57
2	0	0	106	39	145
3	0	0	0	86	86
Total	28	59	119	125	331

**Table 3:** Results of the graded diagnosis of intestinal adhesions on CT with pneumoperitoneum.

The graded diagnosis of intestinal adhesions on CT with pneumoperitoneum	Sensitivity (%)	Specificity (%)	Accuracy (%)
0	100	95.04	95.46
1	74.57	95.22	91.54
2	89.07	81.60	84.29
3	68.80	100	88.21

### Complications of CT pneumoperitoneum

**Figure 5:**
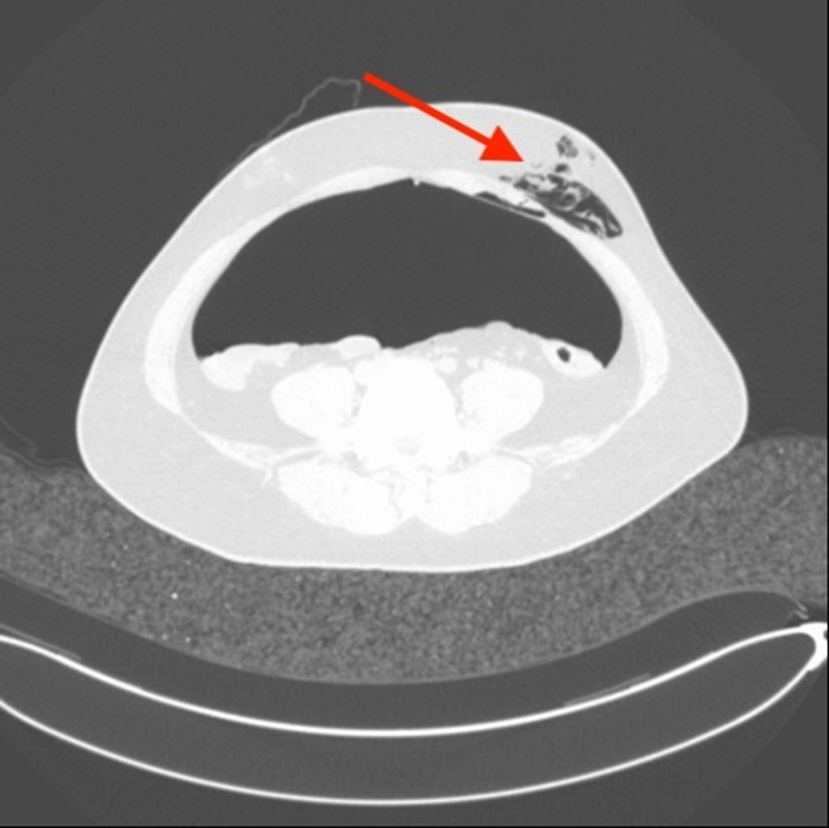
Complications of CT pneumoperitoneum. The arrow shows subcutaneous emphysema in the patient.

In the process of pneumoperitoneum, there are 22 patients developed complications of subcutaneous emphysema (Fig. 5), and the patient recovered well without sequelae in the follow-up treatment.

## Discussion

As early as 1901, Kelling of Germany inserted a cystoscope into the abdomen of a dog for examination and, for the first time, introduced the use of filtered air to create pneumoperitoneum for intra-abdominal examination, called laparoscopic endoscopy^3^. Later, with the development of laparoscopic technology, pneumoperitoneum was used in laparoscopic surgery with the help of gas pressure to separate the abdominal wall from the abdominal organs, providing surgeons with a wide field of vision and an easy-to-operate space^4^. With the rapid development of CT in the twenty-first century, artificial pneumoperitoneum has gradually been used as an imaging method in CT scanning^5–8^. However, most pneumoperitoneal protocols are not standardized. This article aims to explore the standard method and application of pneumoperitoneum in CT through a retrospective analysis of 331 patients with pneumoperitoneum.

### Technical points of artificial pneumoperitoneum

The key step in the angiography process is to determine whether the puncture needle enters the abdominal cavity rather than enters subcutaneously. After the puncture needle enters the abdominal cavity, a small amount of air must first be injected, and then the patient’s pain response should be observed in detail. There should be no resistance when a small amount of air is injected, and the patient should not feel pain. If a small amount of air is injected, the patient may feel that the air has been punctured into the subcutaneous tissue. At this time, pumping will cause subcutaneous emphysema. If there is subcutaneous emphysema, pumping should be stopped immediately and attempted again after the gas is absorbed in 2–3 days. Second, because different patients have different tolerance levels, the time at which and the amount of gas needed to stop pumping cannot be simply judged based on the subjective feelings of the patient. An insufficient pumping volume during pneumoperitoneum will affect the image quality after pneumoperitoneum, making the pneumoperitoneal space impossible to fully reveal. The abdominal organs will also not be clearly separated from the abdominal wall, which will affect the diagnosis.

### The main points of CT scanning technology

When performing CT scanning, attention should be paid to the scanning in the left and right lateral and prone positions. During axial scans, due to gravity and the activity of the abdominal internal organs, the abdominal internal organs are spread horizontally, with a half-moon-shaped pneumoperitoneal space above and the abdominal internal organs located below. At this time, the abdominal internal organs are clearly separated from the anterior abdominal wall. The relationship between the abdominal and posterior abdominal walls on both sides is unclear. If lesions occur in these areas, the horizontal axis cannot be used for diagnosis. Therefore, we believe that it is necessary to scan the patient 4 times, to prevent missed diagnosis. Regarding the radiation dose issue, studies have pointed out that low-dose spiral CT can effectively reduce the radiation dose received by patients while ensuring the quality of image diagnosis^9^. The radiation dose received by the patients in this study was calculated using CTDI. The results showed that the average radiation dose received by the patient after 4 scans was 6.89 mGy, which is much lower than the routine abdominal plain scan dose. The low-dose scanning method can guarantee the patient Security. In this study, we used a smaller tube current method, 35 mA, to reduce the radiation dose received by the patient.

### Application of artificial pneumoperitoneum CT

Artificial pneumoperitoneum CT imaging has good resolution and can observe the relationship between abdominal internal organs and the peritoneum, especially in the diagnosis of abdominal adhesion-related diseases, and it can provide guidance for clinical treatment. The operation is simple, the patient suffers limited pain, and this method is worthy of clinical application.

Among the 331 patients in this study, except for 43 patients with no abnormalities, the remaining 288 patients had abdominal adhesion signs that were confirmed by surgery. Practice has proven that artificial pneumoperitoneum CT has important value in the diagnosis of abdominal adhesions. Since the abdominal wall is separated from the abdominal internal organs in the pneumoperitoneum state, the adhesion between the abdominal internal organs and the abdominal wall can be clearly displayed. However, the adhesion between the intestines is not clear, and it can only be better displayed when the intestinal adhesion is severely adhered to the abdominal wall. This is also the reason for false negatives in the diagnosis results. At the same time, due to the different patient's gas injection volume during pneumoperitoneal CT examination and the patient's breathing movement during scanning, the sensitivity of level 2 diagnosis is higher but the specificity is lower, and the sensitivity of level 3 is higher. Low and high specificity. But overall, the current grading diagnostic standards still have high sensitivity, specificity and accuracy. In future research, we will further refine the criteria for graded diagnosis to provide more accurate diagnosis information.

Compared with transabdominal ultraso- nography (TAU) and cine magnetic resonance imaging (cine MRI)^10^, artificial pneumoperitoneum CT has great potential for improving the accuracy and safety of surgery. Studies have shown that preoperative CT with artificial pneumoperitoneum is helpful for the selection of surgical methods and the formulation of surgical strategies^11^. The pressure of the pneumoperitoneal space established in this research was similar to the pressure of the pneumoperitoneal space established before laparoscopic surgery. Through the use of preoperative artificial pneumoperitoneum in contrast CT and volume rendering technology, the intra-abdominal situation can be visually displayed so that clinicians can have a clear understanding of the patient's condition, and this method may be combined with virtual reality technology for preoperative simulation in subsequent research^12–14^, which will play a more important role in preoperative guidance.

This study retrospectively analysed quantitative artificial pneumoperitoneum cases, summarized the relevant experience, strived to summarize the standardization of related operations and data collection plans, and laid a good foundation for the wide application of artificial pneumoperitoneum CT. This study also has some limitations. Because it is a retrospective study, the data can only be obtained from the patients' past cases, and there may be some errors in the analysis of surgical results and imaging results. For example, the severity of adhesions is simply graded, and the imaging results and surgical results are not accurately compared. This is an unavoidable problem in retrospective research. The formulation of the graded diagnostic criteria of this study still needs to be further refined to facilitate the barrier-free communication with clinicians.

## References

[1] Krielen P, Stommel MWJ, Pargmae P (2020). Adhesion-related readmissions after open and laparoscopic surgery: A retrospective cohort study (SCAR update). Lancet.

[2] Zhao Y, Zhang G, Wang C (2011). Analysis of the research status of abdominal adhesion. J. North China Univ. Sci. Technol. (Health Sci. Ed.).

[3] Yan M (2014). History and expectation of laparoscopic surgery. Mod. Hosp..

[4] Zhang H, Zhang J, Zhou W (2009). Techniques in the establishment of pneumoperitoneum by laparoscopic surgery. J. Kunming Med. Univ..

[5] Cai X, Han C, Ye D (2010). Pneumo-peritoneum helico-CT imaging in post-operative peritoneal adhesion. J. Cent. South Univ. (Med. Sci.).

[6] Yan P, Zhou P, Chen Z (2011). Clinical application of artificial pneumoperitoneum and gastrointestinal contrast CT imaging in diagnosis of abdominal wall adhesion to intestine after operation. Chin. J. Bases Clin. Gen. Surg..

[7] Wan M, Lei L, Zhai C (2018). Application of abdominal computerized tomography in preoperative progressive pneumoperitoneum before hernia repair in patients with large incisional hernia. Chin. J. Hernia Abdom. Wall Surg. (Electron. Ed.).

[8] Cai X, Jia Y, Shi H (2013). Pneumoperitoneum helico-CT imaging for laparoscopic surgery for the patients with previous abdominal operation. Chin. J. Minim. Invasive Surg..

[9] Zhang R, Sun X, Diao J (2010). Application of low dose CT scan in pneumoperitoneal visualization with 64-row multi-slice helical CT. Chin. J. Imaging.

[10] Zinther NB, Fedder J, Friis-Andersen H (2010). Noninvasive detection and mapping of intraabdominal adhesions: A review of the current literature. Surg. Endosc..

[11] Li W, Cao W, Wang X (2019). Progressive artificial pneumoperitoneum before and after 3D accurate volume reconstruction “perspective” abdominal wall hernia before surgery. J. Imaging Res. Med. Appl..

[12] Dawda S, Camara M, Pratt P (2019). Patient-specific simulation of pneumoperitoneum for laparoscopic surgical planning. J. Med. Syst..

[13] Elliott RC, Kirberger RM (2015). Computed tomography determined changes in position of the urogenital system after CO_2_ insufflation to determine optimal positioning for abdominal laparoscopy. Vet. Surg..

[14] Parraga E, Lopez-Albors O, Sanchez-Margallo F (2013). Effects of pneumoperitoneum and body position on the morphology of the caudal cava vein analyzed by MRI and plastinated sections. Surg. Endosc..

